# Power-law coarsening in network-forming phase separation governed by mechanical relaxation

**DOI:** 10.1038/s41467-020-20734-8

**Published:** 2021-02-10

**Authors:** Michio Tateno, Hajime Tanaka

**Affiliations:** 1 Department of Fundamental Engineering, Institute of Industrial Science, University of Tokyo, 4-6-1 Komaba, Meguro-ku, Tokyo, 153-8505 Japan; 2 Graduate School of Arts and Sciences, University of Tokyo, Komaba 3-8-1, Meguro-ku, Tokyo, 153-8902 Japan

**Keywords:** Biophysics, Phase transitions and critical phenomena, Colloids, Fluids

## Abstract

A space-spanning network structure is a basic morphology in phase separation of soft and biomatter, alongside a droplet one. Despite its fundamental and industrial importance, the physical principle underlying such network-forming phase separation remains elusive. Here, we study the network coarsening during gas-liquid-type phase separation of colloidal suspensions and pure fluids, by hydrodynamic and molecular dynamics simulations, respectively. For both, the detailed analyses of the pore sizes and strain field reveal the self-similar network coarsening and the unconventional power-law growth more than a decade according to *ℓ* ∝ *t*^1/2^, where *ℓ* is the characteristic pore size and *t* is the elapsed time. We find that phase-separation dynamics is controlled by mechanical relaxation of the network-forming dense phase, whose limiting process is permeation flow of the solvent for colloidal suspensions and heat transport for pure fluids. This universal coarsening law would contribute to the fundamental physical understanding of network-forming phase separation.

## Abstract (web summary)

The domain growth in phase separation is usually believed to be controlled by material transport. Tateno and Tanaka find that when phase separation causes strong dynamic asymmetry between the two phases, mechanical relaxation dominates and leads to a universally valid scaling law

## Introduction

Phase separation is one of the most fundamental phase transition phenomena and ubiquitous in nature^1,2^. Demixing of oil and water in salad dressing is a typical example. Very recently, the phenomena have attracted considerable renewed interest since the discovery of biological phase separation in living cells^3–6^. In general, phase separation starts from a molecular length scale, and then the characteristic size of phase-separated domains grows with time to a macroscopic scale. Thus, it is crucial to understand how phase-separation morphology is selected and coarsens with time. This problem is significant not only from a fundamental viewpoint^1,2^ but also from an application viewpoint, e.g., the processing of soft materials in electric, medical, cosmetics, paint and food industries^7–10^.

The dynamics of phase separation was studied intensively in the 20th century, and the fundamental physical mechanism was well understood. The scaling concept has been established based on the self-similar growth of the phase-separation pattern, which leads to the power-law growth of the characteristic domain size^1,2^: *ℓ* ∝ *t*^*ν*^ (*t*: time). The exponent *ν* is called the growth exponent, whose value depends on the physical mechanism controlling the coarsening dynamics. It has been well established that for droplet phase separation, the evaporation–condensation or Brownian-coagulation mechanisms (for both, *ν* = 1/3) are relevant, whereas, for bicontinuous phase separation, hydrodynamic coarsening (*ν* = 1) is relevant. These physical mechanisms have successfully described the dynamics of phase separation of various materials with fluidity, ranging from one-component atomic (or molecular) fluids, binary mixtures of simple liquids^1,2,11–16^, to solutions of macromolecules such as polymers, colloids, proteins and emulsions^6,17–20^. These fundamental coarsening laws have tremendously contributed to a broad field of basic science and technology.

Here it should be noted that the above theories were developed for phase separation of fluids taking place near the critical point. In reality, however, phase separation in nature and industrial processes often takes place far from a critical point. Although not widely recognised, this means that the applicability of the above theories can be severely limited. The crucial point is that for deep quench far below the critical point, the difference in the particle density between the two phases becomes significantly large, which may lead to a significant difference in the dynamics between them. Under substantial dynamic difference between the two phases, the slower phase cannot catch up with the speed of domain deformation induced by phase separation, behaving as a viscoelastic body rather than a viscous fluid. Accordingly, the viscoelastic nature of the dense (slower-component-rich) phase plays a critical role in the phase-demixing process, leading to unconventional pattern formation. We called this phenomenon viscoelastic phase separation^21,22^. The most remarkable feature is the formation of the network structure of the minority phase, which was first discovered for polymer solutions^21,22^. In this case, the polymer-specific dynamic effect originating from topological entanglements plays a crucial role and leads to the breakdown of the self-similar domain growth. Since the self-similarity is a prerequisite for the power-law growth, there is no universal coarsening law for the viscoelastic phase separation of polymer solutions.

From this respect, gas–liquid phase separation of single-component atomic (or molecular) fluids may be much simpler than that of polymer solutions because atoms (or molecules) have no (or few) conformational degrees of freedom, unlike polymers, implying a possible self-similar domain growth. The same logic may apply to the solutions of macromolecules with few internal degrees of freedom, such as colloidal suspensions, globular protein solutions, and emulsions. Indeed, we have noticed that for network-forming gas–liquid-type phase separation under deep quench, unconventional coarsening behaviour, i.e., the power-law growth of *ν* = 1/2, has been seen in a variety of systems, including a single-component atomic and molecular system^23–27^ and macromolecular systems (colloidal suspensions^28–31^, protein solutions^32^ and lyotropic liquid crystals^33^). These observations imply a universal physical mechanism behind this unusual power-law growth with the exponent of *ν* = 1/2.

In this work, we aim to reveal the physical mechanism responsible for the unconventional power-law coarsening law of *ℓ* ∝ *t*^1/2^ and how universal it is. To this end, we perform numerical simulations of phase separation in two types of systems. One is gas–liquid phase demixing of a colloidal suspension, and the other is that of a single-component atomic (or molecular) fluid. We find that both systems show the power-law coarsening of the network structures with the exponent of 1/2. We successfully uncover the underlying physical mechanism, in which mechanical relaxation of the dense phase plays a critical role, and establish the universal coarsening law, which is valid for gas–liquid-type phase separation in a variety of materials ranging from pure fluids to soft matter such as colloidal suspensions, protein solutions, and emulsions.

## Results

### Phase separation in dynamically asymmetric systems

**Fig. 1:**
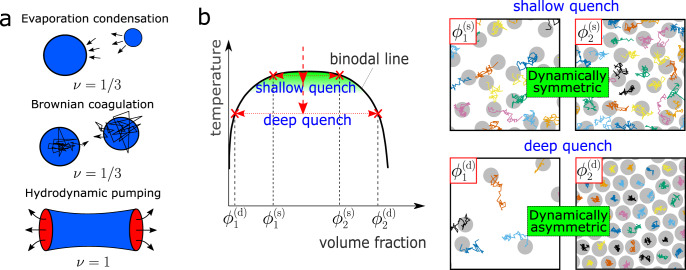
Phase separation and dynamic asymmetry. **a** Three classical coarsening mechanisms for phase separation and the corresponding growth exponents *ν*. **b** The left panel shows a typical phase diagram of colloidal suspensions. For a shallow quench, the two phases have similar relaxation time *τ*_*α*_ because of the similar colloid volume fraction, *ϕ*, between the two phases (see the upper right panel ‘shallow quench’, where we can see similar particle mobilities between the two phases; each particle trajectory is coloured differently). Since the domain deformation rate induced by phase separation is much slower than 1/*τ*_*α*_, both phases behave as viscous liquids. In this case, the growth exponents are those known for classical coarsening mechanisms^17,18,20^. Such behaviours are observed only for a very shallow quench condition (see the green-shaded region; see also Fig. 2). For a deeper quench, on the other hand, the phase with higher *ϕ* has a much slower relaxation time than the one with lower *ϕ* (see the lower right panel ‘deep quench’, where we can see very different particle trajectories between the two phases), and the phase separation proceeds with an average speed between them. As a result, the higher *ϕ* (slower) phase cannot catch up with the domain deformation speed and thus behaves as an elastic body transiently. Thus, the viscoelastic response of the higher *ϕ* phase plays a dominant role in the domain coarsening dynamics, which cannot be described in the conventional theory of phase separation of fluid mixtures. Here we emphasise that this effect is essential in a broad region of the phase diagram in various dynamically asymmetric systems (see Fig. 2).

First, we explain in more detail how the depth of quench affects coarsening process. For a very shallow quench, ordinary phase separation mechanisms are usually valid (see the phase diagram of Fig. 1a and b). For droplet phase separation, the coarsening is driven by the diffusional transport of molecules among droplets (see the top panel of Fig. 1a) or the diffusional transport of droplets and their resulting collision and coalescence (see the middle panel), for both of which *ν* = 1/3. These mechanisms are widely known as the evaporation–condensation (i.e., Lifshitz–Slyozov–Wagner) and the Brownian-coagulation mechanism, respectively^1,2^. For bicontinuous phase separation, material transport is governed by hydrodynamic flow from a narrower part of the network tube to nearby thicker parts due to the Laplace pressure gradient (see the bottom panel of Fig. 1a), and then the tube eventually breaks up, leading to thickening of the nearby tubes. This mechanism is known as Siggia’s hydrodynamic pumping mechanism^34^, whose growth exponent is *ν* = 1.

**Fig. 2:**
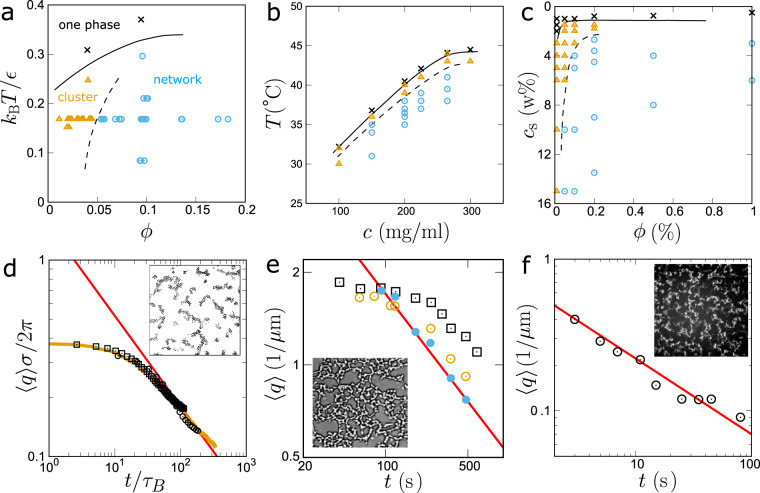
Three different soft-matter systems exhibiting network phase separation with growth exponent *ν*=1/2. **a** A suspension of PMMA colloids^31^, interacting with the depletion attraction of strength, *ϵ*, due to polystyrene added to solvent. *ϕ* represents the colloid volume fraction. **b** Aqueous lysozyme solution^32^, where a temperature quench induces phase separation. *c* represents the protein concentration. **c** A suspension of polystyrene latex colloids^28^, where phase separation is induced by a change of a salt (NaCl) concentration, *c*_*s*_, through an osmotic membrane. *ϕ* represents the colloid volume fraction. The crosses, triangles, and circles represent the state points where we observe a stable one phase, cluster-forming phase separation, and network-forming phase separation, respectively. In panels **a**–**c**, the solid curves are the binodal lines, and the dashed curves show the borders between cluster- and network-forming phase separation. **d** Temporal change of the characteristic wavenumber 〈*q*(*t*)〉 of the phase-separated structure in colloidal systems (see panel **a**) at (*ϕ*, *k*_B_*T*/*ϵ*) = (0.1, 0.17). Time and space are scaled by the colloidal diameter *σ* and Brownian time *τ*_B_, respectively. We show the experimental results of two systems with different *σ*’s, 1.9 μm (circle) and 2.9 μm (square), together with that of the FPD simulation (brown curve). We note that the intercolloid potentials of the three systems are precisely matched. We can see the three curves of 〈*q*(*t*)〉 almost coincide with each other under the above scaling. **e** 〈*q*(*t*)〉 for a lysozyme solution (panel **b**) at *c* = 200 mg/ml and *T* = 36, 37, 38 ^∘^C (square, open circle, filled circle, respectively). **f** 〈*q*(*t*)〉 for latex suspensions (see panel **c**) at (*ϕ*, *c*_*s*_) = (0.1%,10 w%) (circle). The red lines in panels **d**–**f** have a slope of −1/2. The insets of panels **d**–**f** show the network structures observed with microscopy during phase separation (**d** and **f**: the cross-sections of 3D network structures). Note that the characteristic length scale *ℓ* is inversely proportional to 〈*q*(*t*)〉.

For a deep quench far below the critical point, the difference in the particle density between the two phases becomes significantly large (see Fig. 1b), which may lead to a significant difference in the structural relaxation time, *τ*_*α*_, i.e., strong dynamic asymmetry, between them^21,22^. Such a situation is generally realised for gas–liquid-type phase separation under a deep quench (see Fig. 1b). In such a situation, ordinary phase-separation mechanisms do not necessarily work. As we mentioned above, we observed unconventional domain coarsening with the growth exponent *ν* = 1/2. To indicate under what situations this exponent is observed, we show phase diagrams of three different soft-matter systems together with the type of phase separation observed (see Supplementary Note 1 for the details). In ordinary binary liquid mixtures, such as a water-oil mixture, the region where bicontinuous structure appears is limited only to a region where the volume fraction of the minority phase is higher than 32 ± 3%^12^. Contrary to this traditional knowledge, even the minority phase, whose volume fraction is much lower than 30%, forms network structures, instead of droplets, as shown for colloidal suspensions^31^ (a), protein solutions^32^ (b), and charged colloidal suspensions^28^ (c) in Fig. 2. Furthermore, for these systems, the characteristic length of the network, *ℓ*, commonly grows as *ℓ* ∝ *t*^1/2^ in the late stage, while retaining the network connectivity (see Fig. 2d–f). Interestingly, the exponent of *ν* ~ 1/2 has also been observed in the network-forming gas–liquid phase separation of single-component fluids under a deep quench condition^23–27^. This suggests that the coarsening law may be general, but the underlying physical mechanism and its universality have remained elusive.

### Numerical simulations

To reveal the physical mechanism of the peculiar coarsening behaviour, we numerically study phase separation in two systems: gas–liquid phase demixing of a colloidal suspension and that of a single-component atomic (or molecular) fluid. For the former system, we used a hydrodynamic simulation model, fluid particle dynamics (FPD) method^30,35^, which is based on the direct computation of the incompressible Navier–Stokes equation (Supplementary Note 2 for details). We have recently confirmed^31^ that this simulation method can reproduce the colloidal phase-separation kinetics experimentally observed without any adjustable parameters once the interaction potential is matched precisely (see Fig. 2d). In this work, we use a Lennard–Jones (LJ) potential as an interaction potential between colloids since its long-range nature prevents dynamic arrest by gelation. It allows us to access a power-law coarsening regime for nearly two decades. For the latter system, we used standard molecular dynamics (MD) simulations of single-component LJ fluids (see ‘Methods’).

In the following, we first show the numerical simulation results of colloidal suspensions, and then, discuss those of pure atomic fluids, including the similarity and difference between the two.

### Self-similar network coarsening of colloidal phase separation

**Fig. 3:**
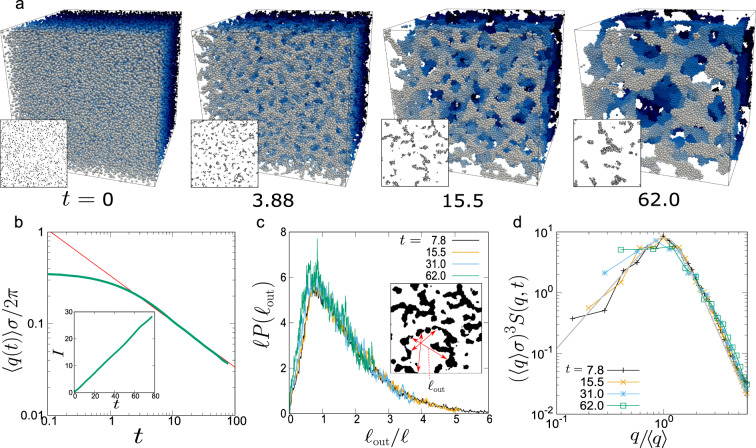
Coarsening behaviour of network-forming phase separation of a colloidal suspension. **a** Time evolution of 3D phase-separation structure (see Supplementary Movie). Particles are coloured to distinguish front particles from back ones. Here we also show the cross-section image of each structure in black&white colour. **b** Temporal change of the characteristic wavenumber 〈*q*〉. The red line represents a power-law function of the exponent −1/2. The inset shows the temporal change of the integrated intensity *I*(*t*) of the structure factor *S*(*q*, *t*). We can see that *I*(*t*) ∝ *t*. **c** The chord length distribution functions for the colloid-poor phase, *P*(*ℓ*_out_), at various times, after scaling the length by the characteristic length *ℓ* = 2*π*/〈*q*〉. *ℓ*_out_ is determine by measuring the length of the lines extending radially from randomly chosen points within the colloid-poor region (see the red arrows in the inset). **d** Scaled structure factors, (〈*q*〉*σ*)^*d*^*S*(*q*, *t*) (*d*: spatial dimension; *d* = 3), as a function of *q*/〈*q*〉 for various times. The grey curve is the fit by the so-called Furukawa function^37^, $\frac{A{x}^{2}}{\frac{\kappa }{2}+{x}^{2+\kappa }}$, where *x* = *q*/〈*q*〉, *κ* = *d* + 1, and *A* is a constant.

We show in Fig. 3a the time evolution of phase-separation structures in a colloidal suspension at the colloid volume fraction of 10% and zero temperature. We can see that a space-spanning network structure of the minority colloid-rich phase spontaneously forms in the early stage, and its characteristic length scale continuously grows with time. In order to characterise the coarsening behaviour quantitatively, we compute the temporal change of the characteristic wavenumber, 〈*q*(*t*)〉 (see ‘Methods’ for its definition), which is inversely proportional to the characteristic length of the network: *ℓ*(*t*) = 2*π*/〈*q*(*t*)〉. As shown in Fig. 3b, we can see a clear power-law coarsening behaviour extending nearly two decades: 〈*q*(*t*)〉 ∝ *t*^−1/2^. We confirm that our results are free from finite-size effects (see Supplementary Note 2, C and Supplementary Fig. 1).

To elucidate which length scale of a real network structure the characteristic length *ℓ* represents, we perform the structural analysis in real space by using the chord length distribution function, *P*(*ℓ*_out_)^36^ (see ‘Methods’ for the details). Organizing the results based on the dynamic scaling concept^1^, we find that the scaling of the length by *ℓ*(*t*) leads to the collapse of the distribution functions *P*(*ℓ*_out_) at various *t* onto a single master curve (see Fig. 3c): *ℓ*(*t*)*P*(*ℓ*_out_) = *f*(*ℓ*_out_/*ℓ*(*t*)), where *f*( ⋅ ) is some function. This result indicates the self-similarity of the coarsening: phase-separation patterns at any time are identical to each other in a statistical sense, once their sizes are scaled by the characteristic lengths *ℓ*(*t*). This fact further indicates the presence of a unique self-similar coarsening mechanism behind this phase-separation process. In Fig. 3c, we can also see that the peak position of the distribution is around *ℓ*_out_(*t*)/*ℓ*(*t*) ~ 0.8, independent of time *t*. It means that the characteristic length, *ℓ*(*t*), obtained by the structure factor, roughly corresponds to the characteristic pore size of the network structure, *ℓ*_out_, at any time. Here we mention that the same scaling law is also valid for the chord length distribution function for the colloid-rich region, *P*(*ℓ*_in_), and its peak is located around *ℓ*_in_/*ℓ*(*t*) ~ 0.2 (Supplementary Note 3 and Supplementary Fig. 2). The fact that the ratio between the characteristic length of the two phases is kept constant with time (i.e., *ℓ*_out_(*t*)/*ℓ*_in_(*t*) ~ 4) is consistent with the self-similar nature of the network growth.

In Fig. 3d, we show that the structure factor, *S*(*q*, *t*) (the definition being described in ‘Methods’) can also be scaled as 〈*q*〉^*d*^*S*(*q*, *t*) = *g*(*q*/〈*q*〉) (*d*: the spatial dimension; *g*( ⋅ ): some function), which is further support for the self-similarity in pattern evolution. We also find that the master curve of *S*(*q*, *t*) can be apparently described by Furukawa’s scaling function^37^: $g(x)\propto \frac{{x}^{2}}{\frac{\kappa }{2}+{x}^{2+\kappa }}$, where *κ* = *d* + 1 (see the grey curve in Fig. 3d), whose low and high *q*-dependences are constrained by the conservation law of the composition and the Porod law due to the sharp domain interface, respectively. Moreover, denoting the *q*-integral of *S*(*q*, *t*) as *I*(*t*), we expect the relation of *I*(*t*) ∝ *ℓ*^*d*−1^ = *ℓ*^2^ for self-similar domain growth^1,2^. In the inset of Fig. 3b, we show that *I*(*t*) indeed linearly increases with time *t* (*I*(*t*) ∝ *t*), which is consistent with the coarsening law of *ℓ* ∝ *t*^1/2^ found in the above.

**Fig. 4:**
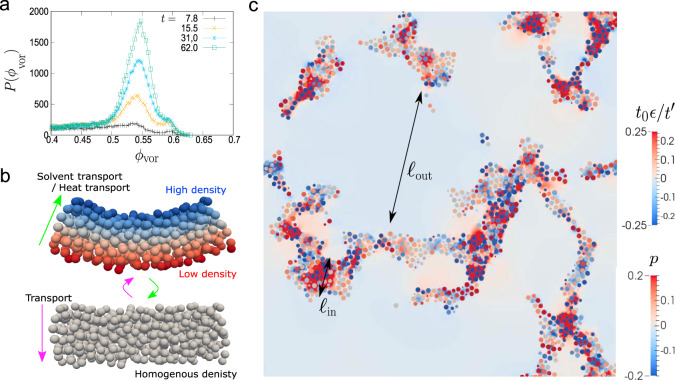
The origin of self-similarity and the key material transport mechanism. **a** The distribution function of the local volume fraction of each particle, ${\phi }_{{\rm{vor}}}=\frac{\pi {\sigma }^{3}}{6{V}_{{\rm{vor}}}}$, where *V*_vor_ is the volume of the Voronoi cell of each particle. In the early stage, since most of the particles are located at the interface of the colloid-rich phase, or having only a few neighbouring particles (~6) (much less than in bulk (~12)), the distribution is very flat. However, in the late stage (*t* ≳ 31.0), we can see a rather sharp peak. The local volume fraction in the colloid-rich phase in the late stage is approximately estimated as *ϕ*_vor_ = 0.54 ± 0.03 and almost constant with time. **b** A schematic illustration of poroelastic and thermoelastic deformations. Elastic volumetric deformation of the dense colloidal domain must involve the solvent transport inside the domain. For example, when the rod-like domain is deformed from the bottom to the top configuration in panel **b**, the colloid volume fraction increases (decreases) in the upper (lower) part of the domain, accompanying the transport of the solvent from the upper to the lower part (see the magenta arrows). For a single-component fluid, on the other hand, heat transport takes place instead of solvent transport, but the underlying physics is to be the same. The green arrows indicate the reverse process. **c** Visualisation of elastic deformation with the solvent exchange by a 2D slice of the phase-separation structure at *t* = 62. The circles represent the cross-sections of colloidal spheres, and the particle colour is labelled according to the scaled local volume deformation introduced in Fig. 5c. The colour in the background (i.e., outside the particles) shows the pressure field of the solvent.

In general, the self-similar nature of the domain coarsening during phase separation is a consequence of the fact that the volume fractions of the two phases keep constant with time, after the formation of a sharp domain interface between them, i.e., the saturation of the compositions of both phases to their equilibrium ones. In Fig. 4a, we indeed find that the colloid volume fraction in the colloid-rich phase *ϕ*, is kept almost constant with time at *ϕ* = 0.54 ± 0.03 in the late stage of phase separation, i.e., in the power-law-growth time regime. We also confirm that the pressure of randomly packed colloids at zero temperature is almost zero at *ϕ* ~ 0.54 (see Supplementary Note 4 and Supplementary Fig. 3). This fact is also consistent with the condition for gas–liquid coexistence (note that since there are few colloids in the colloid-poor phase (see Figs. 3a and 4c), its pressure should be nearly zero).

### Coarsening mechanism

Now, we turn our attention to the coarsening mechanism. In ordinary bicontinuous phase separation^34^, a domain responds as viscous fluid to the mechanical stress generated by the interfacial tension. In other words, both phases behave like viscous fluids. This coarsening mechanism due to hydrodynamic flow leads to the growth exponent of *ν* = 1 (see the right panel of Fig. 1a)^34^, and thus, the domain coarsens much faster than in the present case with *ν* = 1/2. Therefore, it is natural to consider that the viscoelastic nature of the colloid-rich phase may play a critical role in the slower domain coarsening (see the right lower panel of Fig. 1b). The crucial point is that the elastic deformation of the network structure must be accompanied by small local composition change of *δϕ*(**r**) from its average value *ϕ*_0_ ~ 0.54 (Fig. 4a), i.e., the local volume deformation *ϵ*(**r**) = − *δϕ*(**r**)/*ϕ*_0_ (see Fig. 4b). Then, because of the mass conservation law, the volume deformation of the colloid-rich phase must be accompanied by the slow solvent transport, which leads to the permeation of the solvent through small gaps among densely-packed colloids. This situation is precisely that of the poroelastic theory^38^, which, for example, describes slow water transport in soil. We can directly confirm this transport mechanism by looking at the pressure field of the solvent in the colloid-rich domain (see Fig. 4c): The pressure field of the solvent, *p*, is strongly inhomogeneous inside the colloid-rich network, which is coupled with the volume deformation field, *ϵ*. Note that the pressure gradient in the network is the driving force inducing the permeation flow of the solvent. According to the poroelastic theory^38^, the local volume deformation, *ϵ*(**r**, *t*), should obey the following diffusion-type equation:

1 $$\frac{\partial }{\partial t}\epsilon ={D}_{{\rm{P}}}{\nabla }^{2}\epsilon ,$$

where *D*_P_ is the so-called poroelastic diffusivity^38,39^. Here, because the average composition, *ϕ*_0_, inside the colloid-rich phase is almost constant in the coarsening regime (Fig. 4a), the elasticity and permeability of the colloid-rich phase should be more or less constant with time, which allows us to treat *D*_P_ as a constant with time. This result indicates that the mechanical relaxation of the network deformation, which is the crucial process of the network coarsening, is limited by the slow fluid transport through the dense colloid-rich phase (i.e., poroelastic deformation, see Fig. 4b). In other words, the characteristic time of domain deformation is given by the time required for the solvent to transport over the characteristic length *ℓ*. The self-similar nature of the network growth indicates that there is a specific characteristic length for the phase-separation pattern, which is *ℓ*. This fact justifies choosing *ℓ* as the space unit of the Laplacian in front of *ϵ* in Eq. (1) in our scaling analysis. Thus, we obtain the domain coarsening law of $\ell \sim {({D}_{{\rm{P}}}t)}^{1/2}$.

### Evidence for the above mechanism

**Fig. 5:**
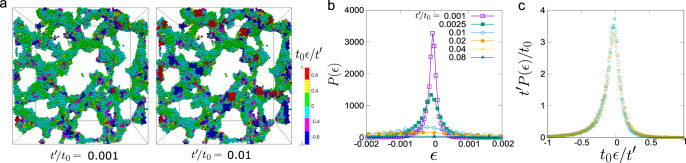
Volume strain in the colloid-rich domain. **a** Real-space mapping of the local volume strain around each particle. Colour labelled on the particles represents the value of the scaled local volume strain ${t}_{0}\epsilon /{t}^{\prime}$. The reference time of the strain is chosen as *t*_0_ = 62.0. Here we show only particles which are located in the top one-quarter of the simulation box to make the details of structures visible. **b** Time evolution of the distribution of local volume strain, *P*(*ϵ*). Here ${t}^{\prime}$ represents the duration of time from the reference time, *t*_0_ = 62.0, to measure the strain. **c** *P*(*ϵ*) after scaling *ϵ* by ${t}^{\prime}/{t}_{0}$. The data are sampled from the data whose reference time is *t*_0_ = 31.0 (blue), 46.5 (green), 62.0 (brown). Then, cross, triangle and square symbols represent the data at ${t}^{\prime}/{t}_{0}=0.001,0.01,0.02$, respectively.

To confirm the validity of the above mechanism, we now focus on the elastic nature of the colloid-rich domain. To this end, we first analyse the strain field, *ϵ*_*αβ*_, by coarse-graining the local displacements of colloidal particles (see ‘Methods’ and Supplementary Note 5 for details). In Fig. 5a, we show the 3D structures of the colloid-rich network together with real-space mapping of local volume strain, *ϵ* (see below). Here we can see that the locations where compression (*ϵ* < 0) or dilation (*ϵ* > 0) takes place are not distributed randomly, but distributed with the characteristic length scale of the network width (i.e., the chord length of the colloid-rich network), *ℓ*_in_. This situation is very similar to the composition and pressure distributions in Fig. 4b, as it should be. Here we note that because of the self-similar nature of the network growth, *ℓ*(*t*) ∝ *ℓ*_in_(*t*). This fact allows us to treat the elastic deformation of the colloid-rich domain in a coarse-grained manner. We also show in Fig. 5b the time evolution of the distribution function, *P*(*ϵ*), of local volume strain with respect to the reference time *t*_0_ (=62.0), $\epsilon ={\sum }_{\alpha }{\epsilon }_{\alpha \alpha }({t}_{0}\to {t}_{0}+{t}^{\prime})$. We note that *t*_0_ = 62.0 corresponds to the right most panel in Fig. 3a. We can see that the peak width broadens and the peak height decreases with the increase of ${t}^{\prime}$. Here we stress that the self-similarity and dynamic scalability hold for the phenomena. Thus, if the elastic response of the colloid-rich phase plays an important role, we expect that the dynamical scaling holds for the distribution function of $P(\epsilon ,{t}_{0},{t}^{\prime})$. Indeed, we find that it can be scaled as $({t}^{\prime}/{t}_{0})P(\epsilon ,{t}_{0},{t}^{\prime})=f({t}_{0}\epsilon /{t}^{\prime})$ (*f*: some function; see Fig. 5c). From this scaling, we may conclude that *ϵ* is proportional to ${t}^{\prime}$ for a certain *t*_0_: $\epsilon \propto {t}^{\prime}/{t}_{0}$. This proportionality can be explained by the linear nature of the Stokes regime: In a short time duration (${t}^{\prime}$), in which the relative displacements between the centre-of-mass positions of colloidal particles are negligibly small compared to the particle size of *σ*, the velocities of colloidal particles should be constant with time. Here, (1) *ϵ* is an infinitesimally small dimensionless quantity, (2) *t*_0_ ∝ *ℓ*^2^, and (3) the above relation of $\epsilon \propto {t}^{\prime}/{t}_{0}$ is to hold for arbitrary ${t}^{\prime}$. These facts (1)–(3) tell us that ${t}^{\prime}$ should also be proportional to *ℓ*^2^. This finding clearly indicates that the growth exponent, *ν* = 1/2, reflects the elastic response inside the colloid-rich phase, whose characteristic time (*τ*_*ϵ*_) and length scales (*ℓ*_*ϵ*_) satisfy ${\tau }_{\epsilon }\propto {\ell }_{\epsilon }^{2}$ (note that *ℓ*(*t*) ∝ *ℓ*_in_(*t*) ~ *ℓ*_*ϵ*_(*t*)). Here we stress that our hydrodynamic simulation method (FPD) strictly satisfies the momentum conservation for colloids and a solvent as well as the incompressibility condition for a solvent, allowing us to observe the local solvent exchange accompanied by subtle volumetric deformation of the colloid-rich domain (see Fig. 4c). This technical feature can generally be attained by simulation methods of colloidal suspensions based on the direct computation of the incompressible Navier–Stokes equations (see, e.g., refs. ^40–42^). In refs. ^31,43^, we discussed the advantages and disadvantages of our simulation method, including comparison with other simulation methods.

Finally, we mention the elementary process of the topological change of the network structure during its coarsening. In the absence of thermal noise (i.e., at zero temperature), the coarsening cannot be due to thermal activation but should be of purely mechanical nature^44^. The network structure is under mechanical stress to reduce the interfacial energy cost. The resulting mechanical stress is concentrated on weak parts of the network structure, leading to their eventual rupture. Then the whole network relaxes its shape while retaining the momentum balance (or, mechanical balance) condition. Such topological change of the network and the resulting slow mechanical relaxation are the primary mechanisms of the network coarsening (see also Supplementary Movie). Since the mechanical rupture is a rapid nonlinear process, it is not the limiting process controlling the network coarsening. It is slow mechanical relaxation that controls the network coarsening. This situation is similar to the case of Brownian-coagulation mechanism for droplet phase separation (see the middle panel of Fig. 1a): slow Brownian motion of droplets is the limiting process of domain coarsening, but rapid droplet coalescence accompanying the topological change is not^1,2,16,34^. The slow mechanical relaxation process described in the above is characterised by the successful scaling of *P*(*ϵ*) with ${\tau }_{\epsilon }\propto {\ell }_{\epsilon }^{2}$, as shown in Fig. 5c.

### Crossover of the limiting transport process

Here we note that the above scenario is valid only when the characteristic time required for poroelastic deformation (i.e., ${\tau }_{\epsilon }={\ell }_{\epsilon }^{2}/{D}_{{\rm{P}}}$) is sufficiently shorter than the structural relaxation time of the colloid-rich phase (*τ*_*α*_). *τ*_*ϵ*_ increases monotonically with domain growth, whereas *τ*_*α*_ is almost constant since it is determined only by the volume fraction of the colloid-rich phase, *ϕ*_0_, which is constant with time (see Fig. 4a). Thus, the above condition is eventually violated in a very late stage. In such a situation, the colloid-rich domains no longer behave as an elastic body and start to behave as a viscous fluid. Then, the domain growth is driven by fluid-like domain deformation, i.e., hydrodynamic transport. Thus, Siggia’s growth exponent (*ν* = 1; see the right panel of Fig. 1a) is to be observed as long as the bicontinuous structure is preserved. Indeed, such a crossover of the growth exponent from 1/2 to 1 was observed in a microgravity experiment carried out in the International Space Station^29^, which successfully followed the phase demixing of colloidal suspensions over five decades. On the other hand, if the connectivity of the network structure is lost, the ordinary mechanisms of droplet growth may start to play a significant role in the domain coarsening.

### Network-forming phase separation in pure fluids

**Fig. 6:**
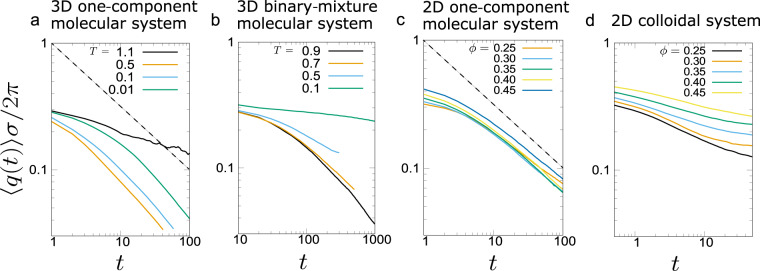
Temporal change of the characteristic wavenumber 〈*q*〉 in phase separation of various systems studied. **a** A single-component fluid system in 3D (*ρ* = 0.33, *T* = 0.01, 0.1, 0.5, 1.1; *ρ*_c_ ~ 0.33, *T*_c_ ~ 1.2). **b** A 50-50 dynamically symmetric mixture of a two-component fluid system in 3D (*ρ* = 1.0, *T* = 0.1, 0.5, 0.7, 0.9; *ρ*_c_ = 0.5, *T*_c_ ~ 1.4). **c** A single-component fluid system in 2D at *T* = 0.3. **d** Colloidal suspensions in 2D at the same parameter setting as in 3D. The dashed lines in panels **a** and **c** have a slope of 1/2.

Next, we turn our attention on network-forming phase separation in single-component fluids. As mentioned above, the domain growth exponent of *ν* ~ 1/2 has also been observed in the gas–liquid phase separation of single-component fluids under a deep quench condition^23–27^. To reveal the underlying mechanism, we study the kinetics of gas–liquid phase separation in a single-component 3D Lennard–Jones (LJ) system (see ‘Methods’ on the simulation details). When the gas and liquid phases have significantly different densities, the two phases exhibit strong dynamic asymmetry (see Fig. 1b). In Fig. 6a, we show the temporal change of the characteristic wavenumber, 〈*q*(*t*)〉, for various quench conditions. We find that 〈*q*(*t*)〉 indeed decays with an almost constant power-law exponent close to 1/2 for a wide range of deep quench conditions (from *T* = 0.5 to 0.01).

**Fig. 7:**
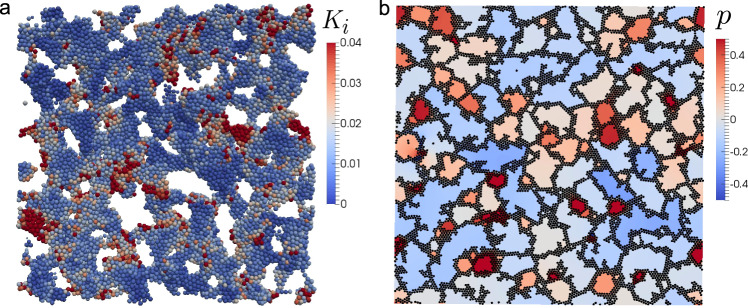
Phase-separation patterns in 3D pure fluid and in 2D colloidal liquid. **a** Real-space mapping of the kinetic energy of each particle *K*_*i*_ in a single-component 3D LJ fluid. Here we show a snapshot at time *t* = 45. The thermodynamic variables are chosen as *ρ* = 0.33 and *T* = 0.01. Particle colour represents the kinetic energy of the particle. **b** Phase separation in 2D colloidal suspension. A snapshot of a phase-separating 2D colloidal suspension (*ϕ* = 38%) at zero temperature at *t* = 500. Here we show the pressure field *p* in the solvent together with the position of colloids. The colour bar represents the pressure.

As in the case of colloidal phase separation, the dense phase is expected to respond elastically to deformation because of its slow dynamics. The domain deformation should be accompanied by small local density change *δρ* around its average density of *ρ*_0_, as in the case of colloidal phase separation (see Fig. 4b). This local density change may further be coupled to the local kinetic energy for a single-component fluid: The more (less) dense the local density is, the less (more) the local kinetic energy is. Note that thermal expansion is the only mechanism of the density change in a single-component system^1^. Then, the relevant transport process should be heat transport. To check whether the coarsening mechanism based on the slow heat transport is relevant or not, we calculate the effective temperature, i.e., the kinetic energy of each particle, ${K}_{i}(t)=\frac{1}{2}m\langle {{\bf{V}}}_{i}^{2}\rangle (t)$, where *i* is the particle index (see ‘Methods’). Figure 7a shows an example of the real-space distribution of *K*_*i*_, where we can see that particles with high/low kinetic energy are not randomly distributed, but heterogeneously with the characteristic length scale of the network structure *ℓ*. This observation supports the mechanism we proposed above.

The pattern in Fig. 7a is quite similar to those shown in Fig. 5a, although the physical quantity displayed is fundamentally different. This similarity in the pattern between the two types of systems can be understood from the similarity between poroelasticity and thermoelasticity: the fundamental equation of poroelasticity^38^ is known to be mathematically equivalent to that of thermoelasticity^45^ (see, e.g., ref. ^39^). This fact indicates that the elastic deformation of the dense phase is limited by the transport of the kinetic energy, i.e., heat transport (see Fig. 4b). Similarly to the case of colloidal phase separation, thus, we have the following diffusion equation for volumetric deformation *ϵ* = − *δρ*/*ρ*_0_ of the network domain:

2 $$\frac{\partial }{\partial t}\epsilon ={D}_{{\rm{T}}}{\nabla }^{2}\epsilon ,$$

where *D*_T_ is the thermal diffusion coefficient and almost constant with time since *ρ*_0_ is almost constant during the coarsening. On noting that the characteristic length is given by *ℓ*, Eq. (2) leads to the domain coarsening law of $\ell \sim {({D}_{{\rm{T}}}t)}^{1/2}$. This relation indicates that the characteristic timescale required for thermal diffusion over the length of *ℓ* is given by ${\tau }_{{\rm{T}}} \sim {\ell }^{2}{D}_{{\rm{T}}}^{-1}$. The thermal diffusion coefficient of the dense glassy system with the similar density *ρ*_0_ is estimated as *D*_T_ ~ 3 from the literature data^46–48^ (see Supplementary Note 6 for details). In Fig. 7a, the characteristic length of domains, or the inhomogeneity of the kinetic energy, is *ℓ* ~ 10 at *t* = 45. Consistently, the above relation provides *τ*_T_ ~ 30 for *ℓ* ~ 10. This result supports the validity of our mechanism.

### Importance of dynamical asymmetry

In the above, we have shown that the power-law growth of the exponent 1/2 in network-forming gas–liquid phase separation of colloidal suspensions and single-component atomic (or molecular) fluids is a consequence of the slow elastic response of the dense phase, whose limiting process is solvent transport (poroelasticity) and heat transport (thermoelasticity) in the dense phase, respectively. Here we examine in more detail under what conditions this coarsening behaviour is to be observed. We need a sufficiently deep quench to induce enough strong dynamic asymmetry between the two phases (see Fig. 1b). However, we note that the growth exponent of 1/2 is not observed for ordinary binary liquid mixtures, where dynamics of the two phases is symmetric. In Fig. 6b, we show the temperature dependence of the coarsening behaviour for a dynamically symmetric binary liquid mixture. We can see that there is no distinct power-law growth, and the apparent growth exponent (i.e., the slope of the curve) continuously decreases with a decrease in temperature. In contrast, in a single-component 3D fluid (Fig. 6a), the characteristic domain size grows more slowly at a lower temperature in the range of 0.5 ≤ *T* ≤ 0.01, yet with the same power-law exponent (*ν* ~ 1/2). The dynamically symmetric binary mixture at the lowest temperature (*T* = 0.1) exhibits a logarithmic-like slow decay of 〈*q*(*t*)〉 (see also ref. ^49^). It is because both phases equally suffer from dynamic arrest due to the glassiness. For such a case, our mechanism is not relevant due to the lack of dynamic contrast between the two phases. For binary mixtures of equal-size particles with dynamic asymmetry, e.g., systems whose components have very different glass-transition temperatures, network phase-separation patterns may be formed. However, the slow inter-species diffusion prevents the early establishment of the saturation of the composition field, which is prerequisite for the scale invariance of the pattern evolution. Thus, we do not expect the power-law domain growth. The absence of the self-similarity and power-law growth was confirmed for polymer mixtures, whose components have very different glass-transition temperatures^50^.

In single-component systems, such a situation never takes place: elastic deformation of the dense phase can proceed without being influenced by the dilute gas phase. It is because the relaxation time of the gas phase is rather insensitive to the temperature and always much faster than the timescale of elastic deformation of the dense liquid phase (see the right panel of Fig. 1b). Thus, a considerable difference in the dynamics (structural relaxation time) between the two phases (i.e., the dilute gas and dense liquid phases) is prerequisite for the power-law growth of exponent 1/2. It is also the case for colloidal suspensions: although a colloidal suspension should be regarded as a binary mixture, the significant size difference between colloids and solvent molecules leads to the strong dynamic asymmetry between the two phases. We may safely assume that the structural relaxation time of a gas (or, solvent-rich) phase is significantly faster than that of liquid (or, colloid-rich) phase (see again the right panel of Fig. 1b). Note that the characteristic timescale of a particulate system is roughly proportional to the cube of the particle size.

In short, our coarsening mechanism is operative in the case where one phase has a space-spanning network structure and exhibits slow elastic motion during coarsening, and the other phase does not hinder the mechanical relaxation process. This situation is widely satisfied with gas–liquid-type phase separation of dynamically asymmetric mixtures.

### Dependence of the growth exponent on the spatial dimensionality

From the above, we may conclude that the growth exponent of 1/2 observed in network-forming phase separation originates from the slow elastic motion of the dense phase under a condition that the other phase does not hinder this process. This condition requires strong dynamic asymmetry between the two phases. This conclusion is valid for three dimensional (3D) systems. However, it may not be necessarily the case for 2D systems. It is because there is an intrinsic topological difference in the percolated network structure between 2D and 3D: in 2D, a bicontinuous network structure can never be formed, unlike in 3D. In the above, we see that for 3D systems, the limiting process of elastic deformation is a slow transport of the solvent or heat, which obeys the diffusion-like equation (Eqs. (1) and (2), respectively). In thermoelasticity, the limiting process is heat transfer, which takes place at any dimension in the same manner. This is confirmed in Fig. 6c: The domain coarsening exponent is *ν* ~ 1/2, even for 2D. In poroelasticity, on the other hand, the limiting process is fluid flow through the dense colloid-rich phase, which obeys Darcy’s law, in which the gradient of fluid pressure induces the flow of the solvent relative to colloids. For a network structure in 2D, the solvent-rich phase cannot have connectivity, and instead, is divided into isolated domains with different pressure, as shown in Fig. 7b. In this situation, isolated solvent-rich domains cannot change their volume easily because of the incompressibility of the solvent, which does not allow the volume deformation of each solvent-rich domain. The only way to change the domain volume is to exchange the solvent between neighbouring solvent-rich domains through the colloid-rich network. This process is very slow. In Fig. 7b, we show a pressure distribution in the solvent-rich domains together with the network of the colloid-rich phase during phase separation of a 2D colloidal suspension. Here we note that the similar pressure field was reported by Yamamoto et al.^51^. The pressure difference between neighbouring domains leads to solvent transport. This transport mechanism imposes a strict boundary condition on the elastic deformation of the colloid-rich network. In Fig. 6d, we show the volume-fraction dependence of the temporal change of the characteristic wavenumber during network-forming colloidal phase separation in 2D. We can see that the growth exponent is much less than 1/2 and strongly depends on the volume fractions, as a consequence of complex nonlocal coupling among solvent-rich isolated domains through a solvent exchange under the constraint of the incompressibility.

The critical point is that pores (i.e., the less dense phase) are isolated for 2D whereas interconnected for 3D. Thus, for 2D colloidal suspensions, permeation flow (Darcy’s law) is induced not only by the pressure gradient inside the percolated network but also by the pressure difference between isolated liquid pores (or, the colloid-poor phase) (see Fig. 7b for the pressure distribution in pores). On the other hand, thermal conduction (Fick’s law) can take place exclusively inside the network for both 2D and 3D single-component fluids since the kinetic energy is inhomogeneous only in the network. Thus, there is no dependence of the coarsening law on the dimensionality for single-component fluids.

### Generality of the coarsening law

Here we discuss for what kinds of systems our coarsening law controlled by mechanical relaxation is relevant.

First, we consider the growth exponent *ν* = 1/2 observed previously by MD simulations of spinodal decomposition. This exponent was reported for 2D gas–liquid spinodal decomposition of single-component fluids^23,52–54^. For this case, the growth exponent of 1/2 was ascribed to the interface-limited (or, ballistic) evaporation–condensation mechanism, where the transport of molecules is kinematic (or, interface-limited) rather than diffusive^1,52,55,56^. The same exponent was also reported for dynamically symmetric binary mixtures^57–60^. In this case, on the other hand, it was ascribed to the Brownian-coagulation mechanism for 2D fluids^61^, in which the Plateau-Rayleigh instability responsible for Siggia’s mechanism in 3D fluids is absent. For both cases, the minority phase forms only droplets unlike our case. Furthermore, the coarsening is governed by thermodynamically-driven transports (ballistic or diffusional) in these mechanisms, whereas by mechanically-driven transport in our mechanism.

The growth exponent suggestive of 1/2 was also reported for the gas–liquid-type spinodal decomposition of 3D pure fluids, based on MD simulations^23–27^. In ref. ^23^, this exponent was ascribed to the interface-limited evaporation–condensation mechanism^52^ for both 2D and 3D. In ref. ^24^, it was speculated that the difference from the coarsening behaviour of the corresponding symmetric binary fluid mixture might be due to the difference in the density and viscosity between the gas and liquid phases, but its exact mechanism has remained elusive. In ref. ^25^, the exponent of *ν* = 1/2 was regarded to be transient before a crossover to ordinary hydrodynamic coarsening with *ν* = 1. In refs. ^26,27^, similarly, it was suggested to be transient before a crossover to faster growth. We speculate that our mechanism may be responsible for these phase-separation behaviours.

For the nucleation-growth-type phase separation with a very asymmetric composition, the same exponent of 1/2 also appears in the time regime where the composition of the majority phase is supersaturated^62–64^. In such a case, diffusional material transport from the surrounding majority phase to droplets of the minority phase controls coarsening dynamics. After the saturation of the majority phase, the evaporation–condensation (or Brownian-coagulation) mechanism starts to play a central role in coarsening (see, e.g., refs. ^63,65^). This mechanism is also governed by thermodynamically-driven transports and nothing to do with our coarsening mechanism, where mechanical relaxation plays a central role.

Finally, we stress that our coarsening mechanism is relevant for phase-separation behaviours observed experimentally for colloidal suspensions and globular protein solutions, as shown in Fig. 2d–f, and also for surfactant solutions^33^. Furthermore, the exponent *ν* = 1/2 was observed over two decades by microgravity experiments of colloidal phase separation^29^. These experimental examples include diverse dynamically asymmetric soft-matter systems with various interparticle potentials, from short-range depletion interaction^29,31^ (Fig. 2d), interprotein interaction^32^ (Fig. 2e), to van der Waals interaction^28^ (Fig. 2f). Moreover, although we consider colloids interacting with the LJ potential in our hydrodynamic simulations, the exponent 1/2 is also numerically reproduced for colloids interacting with short-range depletion attractions^30,31^ (Fig. 2d). These facts indicate the universality of this power-law coarsening with the exponent of 1/2 to a wide variety of network-forming phase separation of dynamically asymmetric mixtures.

## Discussion

In summary, we discover a universal coarsening law (*ℓ* ∝ *t*^1/2^) for gas–liquid-type network-forming phase separation in soft matter and pure fluids, which is valid in a practically relevant condition far from the critical point. We have revealed that the growth exponent of 1/2 is a consequence of the fact that elastic deformation of the dense phase forming a network is controlled by slow transport of the solvent (heat) through it for soft matter (pure fluids). The universality of the coarsening law relies on the absence of complex internal degrees of freedom in the system element, i.e., the presence of a specific length characterising a system; for example, the atomic (or molecular) size in pure fluids^23,26,27^, the colloid size in colloidal suspensions^28–31^, the protein size in protein solutions^32^, and the intermembrane spacing in lyotropic liquid crystals^33^. The mechanism we found here is relevant to gas–liquid-type phase separation of dynamically asymmetric mixtures, in which the coarsening proceeds via solvent (or heat) transport inside the higher-density phase with elasticity. This condition is satisfied for a quite broad class of materials: They include pure atomic and molecular fluids as well as dynamically asymmetric mixtures, e.g., macromolecular systems containing a solvent as the component. We also stress that this mechanism is not restricted to a limited region of the phase diagram but relevant in its broad region (see Figs. 1b and 2). We expect that this mechanism may be relevant to biological phase separation in living cells^3–6^, where various components with different mobility coexist. In this regard, it is notable that very recently, non-spherical network-like morphology has been reported in diverse bio-systems (see, e.g., refs. ^66–68^).

We hope that our finding would contribute to the more profound understanding of phase separation in soft and granular matter, including gel formation^69–71^, as well as in living systems. From an application point of view, our coarsening law would provide a useful guide to design the structures of porous materials, which are used in batteries^72^, ion exchange^9^, catalysis^73^, microelectronics^7^ and medical applications^74,75^.

## Methods

### Simulations of colloidal phase separation

To study colloidal phase separation numerically, we consider a suspension of colloids interacting with LJ potential, $U(r)=4{\epsilon }_{{\rm{LJ}}}\{{(r/{\sigma }_{{\rm{LJ}}})}^{-12}-{(r/{\sigma }_{{\rm{LJ}}})}^{-6}\}$, whose interaction range is much longer than a depletion potential. This long-range nature of the interaction allows us to prevent dynamic arrest due to gelation and thus to follow the coarsening behaviour for a long period. Since our interest is in phase-separation dynamics under a deep quench, we neglect thermal noise. In the data analysis, we use the length unit *σ* as *σ* = *σ*_LJ_ and the time unit *τ*_d_ as *τ*_d_ = 3*πησ*^3^/*ϵ*_LJ_, where *η* is the viscosity of the solvent. *τ*_d_ corresponds to the time during which a free colloid under a constant external force of magnitude, *ϵ*_LJ_/*σ*, moves by its diameter *σ*. The depth of the LJ potential *ϵ*_LJ_ is set such that the Reynolds number $Re=\frac{\rho {\sigma }^{2}}{\eta {\tau }_{{\rm{d}}}}=0.8$ (*ρ* being the density of the solvent). In Supplementary Note 2, B, we confirm the Stokes behaviour for this Reynolds number. We define the volume fraction of colloids, *ϕ*, as $\phi =\frac{\pi {\sigma }^{3}N}{6{L}^{3}}$, where *N* and *L* are the number of colloids and the side length of our cubic simulation box and set *ϕ* = 0.1, which is a volume fraction high enough to form network structures upon phase separation. We perform large-scale FPD simulations with the system size of *L*/*σ* = 69.2 (the corresponding number of the computational grid being 512^3^) by utilising multiple GPUs at the same time.

### Simulations of phase separation of a single-component fluid

To study gas–liquid phase separation, we use a single-component Lennard–Jones system. We utilise the LAMMPS package to perform molecular dynamics simulation with *NVT* ensemble and control the temperature by Nose–Hoover thermostat. We employ the standard Lennard–Jones units (i.e., *σ*_LJ_, ${\tau }_{{\rm{LJ}}}=\sqrt{{\sigma }_{{\rm{LJ}}}^{2}m/{\epsilon }_{{\rm{LJ}}}}$, *ϵ*_LJ_ for length, time and energy units, respectively). We apply the same simulation box size as in the above FPD simulation (*L*/*σ*_LJ_ = 69.2). To simulate gas–liquid phase separation, we first prepare an equilibrium liquid at *ρ* = 0.33 and *T* = 1.8 (the critical number density and temperature being *ρ*_c_ ~ 0.33 and *T*_c_ ~ 1.2, respectively). Then, we quench the system into various temperature, *T* = 1.1, 0.5, 0.1, 0.01 in the unit of *ϵ*_LJ_/*k*_B_, for which we observe the formation of interconnected network structure during phase separation. In the analysis of the local kinetic energy, we calculate the kinetic energy of each particles, ${K}_{i}(t)=\frac{1}{2}m\langle {{\bf{V}}}_{i}^{2}\rangle (t)$ by taking the time average of the velocity of *i*-th particle, **V**_*i*_ over the period $[t-\frac{{\tau }_{{\rm{LJ}}}}{2},t+\frac{{\tau }_{{\rm{LJ}}}}{2}]$. For simulations of a dynamically symmetric binary mixture (see Fig. 6), we employ an LJ potential with the potential depths, *ϵ*_LJ_ and *ϵ*_LJ_/2, for identical and dissimilar particle pairs, respectively (*σ*_LJ_ being common for all pairs of particles).

### Analysis of the temporal growth of the scattering function during demixing

We calculate the scattering function *S*(*q*, *t*) from the 3D power spectrum of the density correlation function as *S*(**q**, *t*) = *ρ*_**q**_(*t*)*ρ*_−**q**_(*t*)/*N*. Here the density field is defined as $\rho ({\bf{r}},t)=\frac{6}{\pi {\sigma }^{3}}{\sum }_{i}{{\Theta }}(\frac{\sigma }{2}-| {\bf{r}}-{{\bf{R}}}_{i}(t)| )$, where Θ is the step function and {**R**_*i*_} is the set of the centre-of-mass positions of colloids. To analyse the temporal change of network patterns during phase separation, we compute the temporal change of the characteristic wavenumber, 〈*q*(*t*)〉, defined as the first moment of the structure factor *S*(*q*, *t*): $\langle q(t)\rangle =\frac{\int dq\,qS(q,t)}{\int dq\,S(q,t)}$, which provides the characteristic wavenumber of a network structure.

### Structural analysis based on the Chord length distribution

To characterise the typical length of the network structure in real space, we use an analysis method called as the chord length distribution^36^. To perform this, we first divide the space into the colloid-rich and poor regions. Specifically, we apply the Gaussian filter with the standard deviation Δ on the centre-of-mass positions of the colloidal particles, and construct a coarse-grained density field, ${\rho }_{{\rm{g}}}({\bf{r}})={\sum }_{i}\exp (-\frac{| {\bf{r}}-{{\bf{R}}}_{i}{| }^{2}}{2{{{\Delta }}}^{2}})$. With this function, we define the part of the space with *ρ*_g_ > *ρ*_th_ (*ρ*_th_ being a threshold value) as the colloid-rich region, and the remaining region as the colloid-poor region. In this analysis, we set as Δ = *σ* and *ρ*_th_ = 1/2. The inset of Fig. 3c shows an example of the binary field obtained by the above procedure. The black and white parts represent the colloid-rich and poor regions, respectively.

The chord length that we use in this paper (*ℓ*_in_ and *ℓ*_out_) is determined in the following way: We randomly choose a point on the space and draw a straight line from the point until the line hitting to the boundary of the colloid-rich and poor regions (see the red arrows in the inset of Fig. 3c). If the chosen point is in the colloid-rich region, we regard the length of the line as *ℓ*_out_; otherwise, as *ℓ*_in_.

### Construction of strain fields

We compute the strain field *ϵ*_*αβ*_, following ref. ^76^. Denoting the displacement of *i*-th particle from time *t* = 0 to *t* = *t* as **u**_*i*_(*t*) = **R**_*i*_(*t*) − **R**_*i*_(0), a coarse-grained displacement field **u**(**r**, *t*) can be written as, ${\bf{u}}({\bf{r}},t)=\frac{{\sum }_{i}{{\bf{u}}}_{i}(t)G({\bf{r}}-{{\bf{R}}}_{i}(t))}{{\sum }_{i}G({\bf{r}}-{{\bf{R}}}_{i}(t))},$ where *G*(**r**) is a coarse-grain function and we employ the following Gaussian form: $G({\bf{r}})=\frac{1}{{(\sqrt{\pi }\sigma )}^{3}}\exp (-\frac{{{\bf{r}}}^{2}}{{\sigma }^{2}})$. The strain field *ϵ*_*αβ*_ is defined as ${\epsilon }_{\alpha \beta }({\bf{r}},t)=\frac{1}{2}(\frac{\partial {u}_{\alpha }({\bf{r}},t)}{\partial {r}_{\beta }}+\frac{\partial {u}_{\beta }({\bf{r}},t)}{\partial {r}_{\alpha }}).$

## Supplementary information

Supplementary Information

Description of Additional Supplementary Files

Supplementary Movie

## Data Availability

The data that support the findings of this study are available from the corresponding author upon reasonable request.

## Code availability

The codes that are used to generate results in the paper are available from the corresponding author upon reasonable request.
