# Supplementary material for: Power-law coarsening in network-forming phase separation governed by mechanical relaxation
Source: Nat Commun. 2021 Feb 10;12:912. doi: 10.1038/s41467-020-20734-8 (PMC7875975; doi:10.1038/s41467-020-20734-8)
Supplement: Supplementary file 1 — Supplementary Information [file 41467_2020_20734_MOESM1_ESM.pdf]

Supplementary information for "Power-law coarsening in network-forming phase separation governed by mechanical relaxation"

Michio Tateno[1,2] and Hajime Tanaka[1]

[1] *Department of Fundamental Engineering, Institute of Industrial Science, University of Tokyo, 4-6-1 Komaba, Meguro-ku, Tokyo 153-8505, Japan*

[2] *Graduate School of Arts and Sciences, University of Tokyo, Komaba 3-8-1, Meguro-ku, Tokyo 153-8902, Japan*

Supplementary Note 1: Summary of experimental observation of network-forming phase separation in soft matter

In Figs. 2a-c, we show phase diagrams of three different dynamically asymmetric mixtures together with the type of phase separation observed (cluster or network): (a) a mixture of PMMA (polymethyl methacrylate) colloids + a solvent with polystyrene as a depletant; (b) an aqueous globular protein (lysozyme) solution; (c) a mixture of polystyrene latex colloids and water (an aqueous suspension of charged colloids). In system (a), the colloids interact with short-range depletion interaction (the range of the interaction being 0.12 times the diameter of the colloids). The control parameter of the system is the volume fraction of the colloids, ϕ, and the temperature scaled by the attraction well depth, $k_{\mathrm{B}}T/\epsilon$ (ϵ being controlled by the concentration of the depletant). The corresponding experimental phase diagram is shown in Fig. 2a. In systems (b) and (c), particles interact with van der Waals attraction and electrostatic repulsion. In system (b), we add 7.5 w% of salt (NaCl) to screen electrostatic interaction between proteins, which sets the critical point slightly above the room temperature. The control parameters are the concentration of the proteins, c, and temperature, T (see Fig. 2b). In system (c), on the other hand, we fix the temperature at the room temperature and control the volume fraction of the colloids, ϕ, and the concentration of salt (NaCl), c_{s} (see Fig. 2c). See Refs. [1], [2] and [3] on the detailed descriptions of systems (a), (b), and (c), respectively.

In ordinary binary liquid mixtures such as a water-oil mixture, the region where bicontinuous structure appears is limited only to a region where the volume fraction of the minority phase is higher than $32 \pm 3\%$ [4]. Contrary to this traditional knowledge, even the minority phase, whose volume fraction is less than $\sim 30\%$, forms network structures, instead of droplets, for phase separation of complex fluids as shown in Figs. 2a-c (see also the insets of Figs.2d-f). We emphasise that the ordinary droplet-type phase separation is observed only for shallow quenches, i.e., in the region between the solid and dashed curves in the phase diagrams of Figs. 2a-c.

Supplementary Note 2: Simulation method for colloidal suspensions

A. FPD method

In order to deal with the hydrodynamic degrees of freedom of colloidal suspensions by numerical simulation, we need to solve the moving boundary problem for all colloidal particles if we treat colloids as solids since the non-slip solid-fluid boundary condition must be satisfied on the surfaces of all colloid particles in motion. The fluid-particle-dynamics method (FPD) [5, 6] regards colloidal particles as undeformable fluid particles whose viscosity is much higher than the solvent viscosity and the viscosity changes smoothly across the colloid-solvent boundary. These features allow us to treat the dynamics of colloids, including many-body hydrodynamic interactions without suffering from the solid-fluid boundary condition, only by solving the incompressible Navier-Stokes equation in a Cartesian coordinate system.

We express the center-of-mass position and velocity of colloid $n(=0,1,..,N-1)$ as $\mathbf{R}_n(t)$ and $\mathbf{V}_n(t)$ respectively, and the force acting on colloid n as $\mathbf{F}_n(t)$. To smoothly connect the viscosity between the colloids and the solvent, we introduce the particle field defined as

$$\phi(\mathbf{r}) = \sum_{n=0}^{N-1} \phi_n(\mathbf{r}),$$

$$\phi_n(\mathbf{r}) = \frac{1}{2}\{\tanh\{(a - |\mathbf{r} - \mathbf{R}_n|)/\xi\} + 1\}.$$

Then the viscosity field is given by

$$\eta(\mathbf{r}) = \phi(\mathbf{r})\eta_{\mathrm{c}} + (1 - \phi(\mathbf{r}))\eta_s,$$

where a is the colloid radius, ξ is the thickness of the smooth interface between the colloids and the solvent, and η_{s} and η_{c} are the viscosity of the solvent and the one inside colloids respectively. Then, we treat the motion of colloid n as a solid body, by expressing its center-of-mass velocity by the spatial average of the velocity field inside the colloid as

$$\frac{d\mathbf{R}_n(t)}{dt} = \mathbf{V}_n(t) = \frac{\int \mathbf{v}(\mathbf{r})\phi_n(\mathbf{r})d\mathbf{r}}{\int \phi_n(\mathbf{r})d\mathbf{r}}, \tag{1}$$

where $\mathbf{v}(\mathbf{r})$ is the fluid velocity field at $\mathbf{r}$ and its time evolution follows the Navier-Stokes equation for the incompressible fluid:

$$\rho(\frac{\partial}{\partial t} + \mathbf{v}\cdot\nabla)\mathbf{v} = \mathbf{f} + \nabla\cdot(\boldsymbol{\sigma}^{\mathrm{I}} + \boldsymbol{\sigma}^{\mathrm{R}}), \tag{2}$$

and

$$\nabla \cdot \mathbf{v} = 0. \tag{3}$$

Here $\boldsymbol{\sigma}^{\mathrm{I}}$ is the internal stress of fluid which is expressed as

$$\boldsymbol{\sigma}^{\mathrm{I}} = \eta(\mathbf{r})(\nabla\mathbf{v} + (\nabla\mathbf{v})^{\mathrm{T}}) - p\boldsymbol{I},$$

where p is the pressure and determined to satisfy the incompressible condition. $\boldsymbol{\sigma}^{\mathrm{R}}$ is the random fluctuating stress field and obeys the following fluctuation dissipation relation:

$$\langle \boldsymbol{\sigma}^{\mathrm{R}} \rangle = \mathbf{0},$$

and

$$\langle \sigma^{\mathrm{R}}_{ij}(\mathbf{r}, t)\sigma^{\mathrm{R}}_{kl}(\mathbf{r}', t')\rangle = 2\eta(\mathbf{r})k_{\mathrm{B}}T(\delta_{ik}\delta_{jl} + \delta_{il}\delta_{jk})\delta(\mathbf{r} - \mathbf{r}')\delta(t - t').$$

Then, $\mathbf{f}$ represents the body force field acting on the fluid, which is obtained from the force acting on colloid n, $\mathbf{F}_n(t)$, as follows,

$$\mathbf{f}(\mathbf{r}) = \sum_n \frac{\mathbf{F}_n \phi_n(\mathbf{r})}{\int \phi_n(\mathbf{r}')d\mathbf{r}'}. \tag{4}$$

We set the lattice unit l as $l = \xi$ and the time unit τ as $\tau = \rho l^2/\eta_{\mathrm{s}}$, which is the time required for the momentum to diffuse over the lattice size. We also set the mass unit as ρl^3. In what follows, we scale all physical variables by the units defined above. In this work, we set $\eta_{\mathrm{c}}/\eta_{\mathrm{s}} = 50$ and $a = 3.2$. The time step used in our simulation is $\Delta t = 0.0025$.

To study the dynamics of colloidal phase separation numerically, we consider a suspension of colloids interacting with a Lennard-Jones (LJ) potential, $U(r) = 4\epsilon_{\mathrm{LJ}}\{(r/\sigma_{\mathrm{LJ}})^{-12} - (r/\sigma_{\mathrm{LJ}})^{-6}\}$. In the data analysis, we set the length unit σ as $\sigma = \sigma_{\mathrm{LJ}}$ and the time unit τ_{d} as $\tau_{\mathrm{d}} = 3\pi\eta_{\mathrm{s}}\sigma^3/\epsilon_{\mathrm{LJ}}$. τ_{d} is the time required for an isolated colloid to move by its diameter σ under an action of the constant external force of amplitude $\epsilon_{\mathrm{LJ}}/\sigma$. We set the depth of the LJ potential ϵ_{LJ} such that the Reynolds number is to be $Re = \frac{\rho\sigma^2}{\eta\tau_{\mathrm{d}}} = 0.8$ (ρ being the density of the solvent). We confirm that the inertia term is negligible and the Stokes behaviour is observed with this Reynolds number, as will be shown below.

As we are interested in the coarsening process of deeply-quenched colloidal phase separation, we perform FPD simulations without thermal noise (i.e., $T = 0$) under a periodic boundary condition. To examine the finite size effects, we employ simulation boxes with three different sizes: $L^3 = 128^3, 256^3$ and 512^3 (or, $(L/\sigma)^3 = 17.3^3, 34.6^3$ and 69.2^3). We set

the volume fraction of the system defined as $\phi = \frac{N\pi\sigma^3}{6L^3}$ as $\phi = 0.1$, where we can see the network-forming phase separation.

Our FPD method is a hybrid simulation method combining lattice simulation for fluid dynamics $\mathbf{v}$ and off-lattice simulation for particle motion $\mathbf{V}_n$. Initially, particles are distributed randomly in space while avoiding the overlap between them. Then we solve the above equation by the Marker-and-Cell (MAC) method with a staggered lattice under the periodic boundary condition. Under the force field $\mathbf{f}$ determined by the particle distribution, we calculate the velocity field by using Eq. (2). By calculating the centre-of-mass velocity of each particle by Eq. (1), we move particles by $\Delta\mathbf{R}_n = \mathbf{V}_n\Delta t$, where Δt is a time step, off the lattice. Then we map all the particles, which are treated off the lattice, on the lattice and solve Eq. (2) to obtain a new flow field $\mathbf{v}$ with the new particle distribution. We repeat this procedure to follow the dynamics. In this way, we can simulate the phase-separation dynamics of colloidal suspensions while including interparticle hydrodynamic interactions.

B. Reynolds number in our simulation

As explained in the previous section, in our FPD simulation, we compute the Navier-Stokes equation, including the inertia term. This term can usually be neglected when we study spontaneous structural formation in colloidal suspensions, which is widely known as the Stokes approximation. In our study, as we are interested in aggregation kinetics of colloids with a sufficiently deep quench, we neglect thermal noise in our hydrodynamic model (or set $T = 0$). Thus, Reynolds number $Re = \frac{\rho\sigma^2}{\eta\tau_{\mathrm{d}}}$ is the only parameter to determine the balance between the various terms in the Navier-Stokes equation, and the Stokes approximation should be valid if Re is sufficiently small. In this study, we set $Re = 0.8$. Now we check whether our simulations are in the Stokes regime under this parameter setting.

To examine this point, we consider the motion of a free colloid. Assuming that a constant force F_{d} is applied to the particle at time $t = 0$, the velocity of the particle V can be written as

$$M_{\mathrm{eff}}\frac{dV}{dt} = F_{\mathrm{d}}\psi^{\mathrm{T}}(\frac{t}{\tau_{\mathrm{a}}}) \tag{5}$$

where $M_{\mathrm{eff}} = \frac{3}{2}M$ is the effective mass of the colloid. Then, $\psi^{\mathrm{T}}(\frac{t}{\tau_{\mathrm{a}}})$ is the memory function

for translational motion that takes hydrodynamic effects into account, and given as [7]

$$\psi^{\mathrm{T}}(x)= \frac{\alpha_+ w(i\alpha_+\sqrt{x}) - \alpha_- w(i\alpha_-\sqrt{x})}{(\alpha_+ - \alpha_-)}, \tag{6}$$

where w is the complex complementary error function, $\alpha_\pm = \frac{3}{2} \pm \frac{\sqrt{3}}{2}i$. The asymptotic behaviour of ψ^{T} in the large x (or, long-time) limit are given by $\psi^{\mathrm{T}}(x) \sim \frac{1}{6\sqrt{\pi}}x^{-3/2}$, which is widely known as a long-time tail. τ_{a} is the time required for the momentum to diffuse over the radius of the colloid and given as $\tau_{\mathrm{a}} = (\frac{\sigma}{2})^2/\nu$, where $\nu = \eta/\rho$ is the kinetic viscosity. We note that τ_{a} is related to τ_{d} as $\tau_{\mathrm{a}} = Re\ \tau_{\mathrm{d}}/4$.

At the timescale $t \sim \tau_{\mathrm{d}}$, ψ^{T} can be estimated as $\psi^{\mathrm{T}}(4Re^{-1}) = \psi^{\mathrm{T}}(5) \sim 0.0076$. The ratio between the magnitude of the momentum of the particle and that of the force is given by

$$\frac{M}{F_{\mathrm{d}}}\frac{dV}{dt} = \frac{2}{3}\psi^{\mathrm{T}}, \tag{7}$$

which is estimated as ~ 0.0051, allowing us to assume that the force acting on the particle is almost perfectly balanced with the viscous drag force, i.e., the inertia term can be neglected.

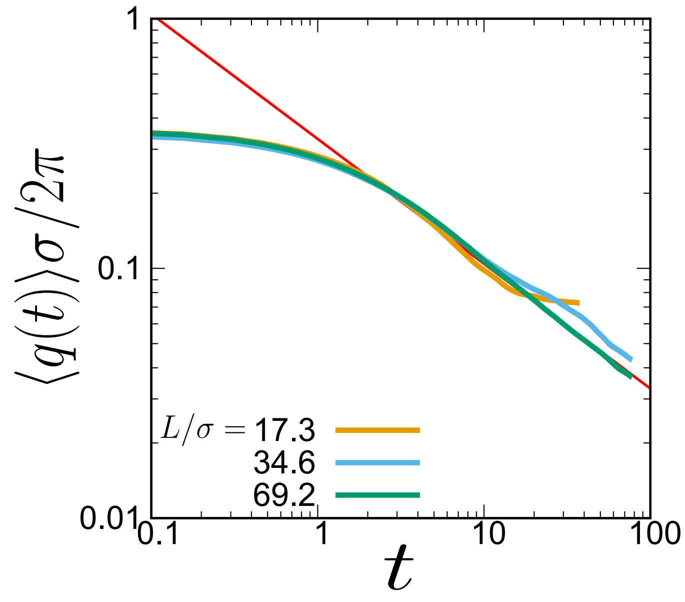

Supplementary Figure 1: Temporal change of the characteristic wave number $\langle q\rangle$ for three different simulation box sizes, $L/\sigma = 17.3, 34.6, 69.2$. The red line represents a power law of the exponent -1/2.

C. Finite size effects

To check whether our simulation box is large enough to study the coarsening behaviour without suffering from a finite-size effect, here we perform the data analysis for simulation results for three different sizes of the simulation box, $L/\sigma = 17.3, 34.6, 69.2$. As shown in Supplementary Figure 1, all the results show almost identical coarsening behaviour: $\langle q \rangle \propto t^{-1/2}$. At the same time, however, we can see that $\langle q(t) \rangle$ deviate from the power law in the late stage for $L/\sigma = 17.3, 34.6$, whereas for $L/\sigma = 69.2$ it continues to obey the power-law over the entire time range of the simulation. It tells us that the deviation from the power-law decay is because of finite size effects. In the main text, we only discuss the simulation results from the largest system size.

Supplementary Note 3: Scaling analysis based on the chord length distribution

In Fig. 3c, we have shown that the chord length distribution for the colloid-poor region $P(\ell_{\rm out})$ can be scaled by the characteristic length scale of the network domain ($\ell = 2\pi/\langle q \rangle$). In Supplementary Figure 2, we also show the chord length distribution for the colloid-rich region. We can see that $P(\ell_{\rm in})$ at different times can be collapsed onto a single master curve, after the same scaling procedure. There is a small difference in the time from which the dynamic scaling becomes valid, between $P(\ell_{\rm out})$ and $P(\ell_{\rm in})$. This simply comes from the difference between the sizes of the colloid-poor ($\ell_{\rm out}$) and colloid-rich domains ($\ell_{\rm in}$) relative to the colloid size σ: for example, the time when the peak position of the distribution becomes $\sim 3\sigma$ is around $t \sim 15.5$ and $t \sim 1.56$ for $P(\ell_{\rm out})$ and $P(\ell_{\rm in})$, respectively.

Additionally, we mention the correspondence of the above analysis to the volume fraction of two phases. Denoting the volume fraction of colloids in the total system and that in the colloid-rich phase as $\phi_{\rm tot}$ and $\phi_{\rm in}$, we may write the volume fraction of the colloid-rich phase Φ as $\Phi = \phi_{\rm tot}/\phi_{\rm in}$. Since we set $\phi_{\rm tot} = 0.10$ in our simulation, and from Fig. 4a we find $\phi_{\rm in} \sim 0.54$, we obtain $\Phi \sim 0.19$. On the other hand, from the analysis based on the chord

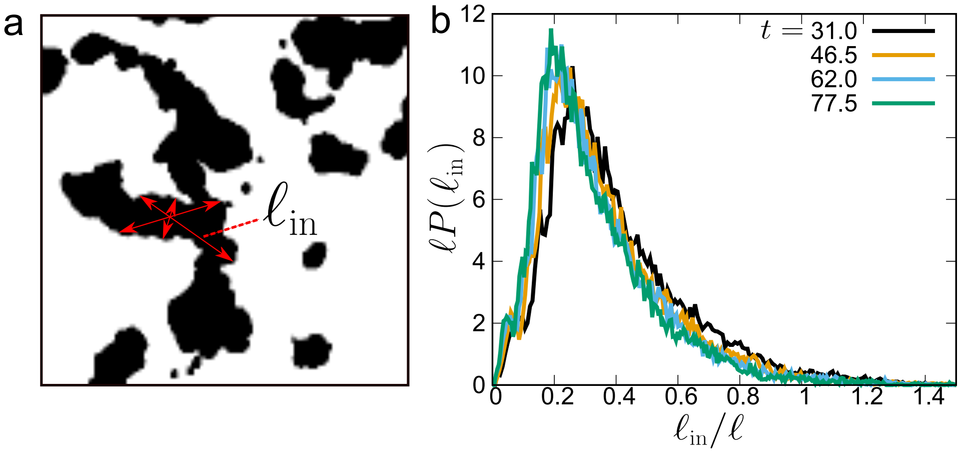

Supplementary Figure 2: **Chord length distribution of the colloid-rich domains. a,** Chord length analysis for the colloid-rich domains. The black regions show cross sections of the colloid-rich domains, and the remaining white part represents the colloid-poor domains. **b,** Chord length distribution for the colloid-rich domains. $\ell_{\rm in}$ is determined by measuring the length of the line extending radially from randomly chosen points with in a colloid-rich region (see the red arrows in panel a).

length distribution, we find that the peak positions of the distributions are located around $\ell_{\rm in}/\ell \sim 0.2$ and $\ell_{\rm out}/\ell \sim 0.8$. It indicates that the phase separation proceeds while retaining the volume ratio between the colloid-rich and poor phases with approximately 20:80, which is consistent with the value obtained in the above ($\Phi \sim 0.19$).

Supplementary Note 4: Volume fraction dependence of the pressure for randomly packed colloids at zero temperature

In Supplementary Figure 3, we show the volume fraction dependence of the pressure for randomly packed colloids at zero temperature. To create a randomly packed configuration, we use the steepest descent technique and measure the pressure by virial stress. The pressure obtained is scaled by the Lennard-Jones units. Here we can see that the pressure has a value close to zero at $\phi \sim 0.54$, indicating that the condition for a gas-liquid coexistence is indeed satisfied. Note that the dense colloid-rich phase has the volume fraction $\phi \sim 0.54$ in the late stage of phase separation (see Fig.4a).

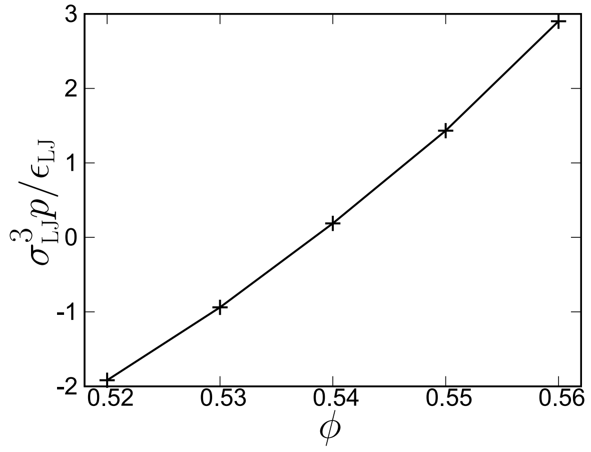

Supplementary Figure 3: **Volume-fraction dependence of the pressure for randomly packed colloids at zero temperature.** Cross symbols represent pressure p scaled by the Lennard-Jones units for five different volume fractions ϕ. The pressure crosses zero at $\phi \sim 0.54$, which is consistent with the peak position of Fig. 4a.

Supplementary Note 5: Analysis of strain fields

A. The distribution of shear strain

We calculate the distribution of shear strain, $P(\gamma)$, for various reference time t_0 and elapsed time t'. Here we define the shear strain as $\gamma = \frac{1}{3}(\epsilon_{xy} + \epsilon_{yz} + \epsilon_{zx})$. Supplementary Figure 4 shows $P(\gamma)$ under the same scaling operation applied to the volume strain (see the main text), i.e., $t_0 P(\gamma)/t' = f(t'\gamma/t_0)$.

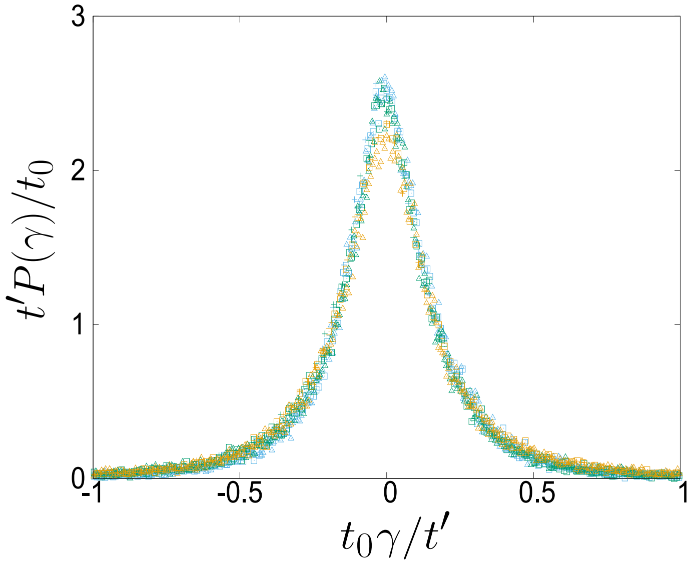

Supplementary Figure 4: **Shear strain distribution,** $P(\gamma)$**, after scaling** γ **by** t'/t_0**.** The data are taken from the data whose reference time is $t_0 = 31.0$ (blue), 46.5 (green), and 62.0 (grown symbols). Cross, triangle and square symbols correspond to the data at $t'/t_0 = 0.001$, 0.01, and 0.02, respectively.

B. Break down of the scaling of volume strain over a long time

In Supplementary Figure 5, we show the scaled distribution of volume strain for a wide range of time. Here we can see that the scaled distributions show almost identical functional form for the early time regime. However, from around $t'/t_0 \sim 0.04$ the data start to deviate from the master curve, suggesting that for such a long period the deformation of the colloid-

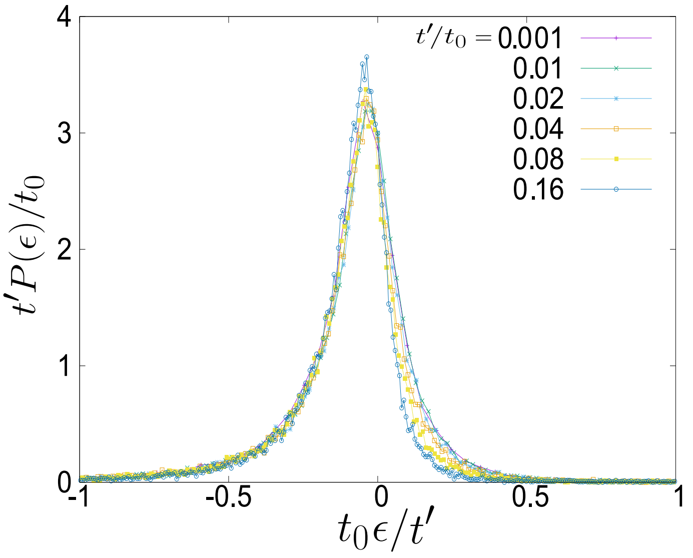

Supplementary Figure 5: **Volume strain distribution,** $P(\epsilon)$**, after scaling** ϵ **by** t'/t_0**.** The reference time is set as $t_0 = 62.0$.

rich domain cannot be described by a linear elastic theory anymore, and nonlinear effects come into play.

C. A decomposition of volume strain along the principle axis

To see the behaviour of volume strain in more detail, we compute the eigenvalues of strain tensor $\epsilon_{\alpha\beta}$, ϵ_i ($i = 1, 2, 3$; $\epsilon_1 \leq \epsilon_2 \leq \epsilon_3$). The sum of these eigenvalues corresponds to the volume strain ϵ. Supplementary Figure 6 shows the distributions of ϵ_i, and we can see the three distinct populations. Furthermore, interestingly we can also see that they are distributed such that $\epsilon_1 \leq 0 \leq \epsilon_3$, clearly indicating that the colloid-rich domains cannot compress (or expand) in all the three directions at the same time. It indicates that the solvent hinders the homogeneous volumetric deformation of colloids, which is consistent with what the poroelastic theory tells us.

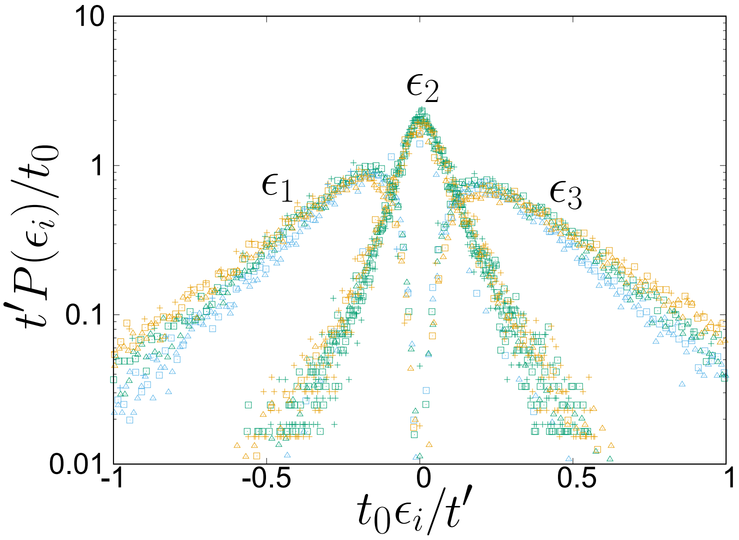

Supplementary Figure 6: **The distribution of the volume strain component** i**,** $P(\epsilon_i)$ **after scaling** ϵ_i **by** t'/t_0**.** The data are taken from the data whose reference time is $t_0 = 31.0$ (blue), 46.5 (green), and 62.0 (grown symbols). Cross, triangle and square symbols correspond to the data at $t'/t_0 = 0.001$, 0.01, and 0.02, respectively.

Supplementary Note 6: Estimation of the thermal diffusion coefficient for a Lennard-Jones glass

Here we estimate the thermal diffusion coefficient D_{T} of the dense network-forming phase from the literature data, by using the relation of $D_{\mathrm{T}} = \frac{\lambda}{\rho C_p}$, where ρ, λ, and C_p is the number density, thermal conductivity and heat capacity at constant pressure, respectively. In the network-forming phase separation of a pure fluid, the densely-packed glassy phase coexists with the gas phase with almost no particles. Thus, the thermal diffusion in the network should almost be equivalent to that in a Lennard-Jones glass at zero pressure. According to Refs. 8 and 9, $\lambda \sim 9.52$, $\rho \sim 1.03$, and $C_p \sim 3.0$ in the Lennard-Jones units for such a Lennard-Jones glass. Thus, we obtain $D_{\mathrm{T}} \sim 3$. We note that in a classical system without quantum effects, these quantities are temperature-independent in the low-temperature region we focus on (see, e.g., Ref. 10).

SUPPLEMENTARY REFERENCES

[1] Tateno, M. & Tanaka, H. Numerical prediction of colloidal phase separation by direct computation of navier–stokes equation. *npj Comput. Mater.* **5**, 40 (2019).

[2] Tanaka, H. & Nishikawa, Y. Viscoelastic phase separation of protein solutions. *Phys. Rev. Lett.* **95**, 078103 (2005).

[3] Tanaka, H., Nishikawa, Y. & Koyama, T. Network-forming phase separation of colloidal suspensions. *J. Phys.: Condens. Matter* **17**, L143 (2005).

[4] Beysens, D. A. Kinetics and morphology of phase separation in fluids: The role of droplet coalescence. *Physica A* **239**, 329–339 (1997).

[5] Tanaka, H. & Araki, T. Simulation method of colloidal suspensions with hydrodynamic interactions: Fluid particle dynamics. *Phys. Rev. Lett.* **85**, 1338 (2000).

[6] Furukawa, A. & Tanaka, H. Key role of hydrodynamic interactions in colloidal gelation. *Phys. Rev. Lett.* **104**, 245702 (2010).

[7] Hinch, E. J. Application of the langevin equation to fluid suspensions. *J. Fluid Mech.* **72**, 499–511 (1975).

[8] Clarke, J. H. Molecular dynamics studies of glass formation in the lennard-jones model of argon. *J. Chem. Soc., Faraday Trans. 2* **75**, 1371–1387 (1979).

[9] McGaughey, A. & Kaviany, M. Thermal conductivity decomposition and analysis using molecular dynamics simulations. part i. lennard-jones argon. *Int. J. Heat Mass Transfer* **47**, 1783–1798 (2004).

[10] Mizuno, H., Mossa, S. & Barrat, J.-L. Elastic heterogeneity, vibrational states, and thermal conductivity across an amorphisation transition. *Europhys. Lett.* **104**, 56001 (2013).
