# Supplementary material for: Power-law coarsening in network-forming phase separation governed by mechanical relaxation
Source: Nat Commun. 2021 Feb 10;12:912. doi: 10.1038/s41467-020-20734-8 (PMC7875975; doi:10.1038/s41467-020-20734-8)
Supplement: Supplementary file 2 — Description of Additional Supplementary Files [file 41467_2020_20734_MOESM2_ESM.pdf]

Description of Additional Supplementary Files

File Name: Supplementary Movie 1
Description: The movie shows the time evolution of the 3D phase-separation structure of a colloidal suspension computed by FPD simulation (see Fig. 3a). The box size and volume fraction are set as $\phi = 0.1$ and $L^3 = (69.2\,\sigma)^3$, respectively. The unit of the time label shown in the movie is chosen as t_{d}. Particles are coloured to distinguish front particles from back ones.
